# Conservative management for patients with chronic kidney disease refusing dialysis

**DOI:** 10.1590/2175-8239-JBN-2018-0028

**Published:** 2018-07-23

**Authors:** Manuel Carlos Martins Castro

**Affiliations:** 1 Instituto de Nefrologia de Taubaté e São José dos Campos, Taubaté, SP, Brasil.

**Keywords:** Chronic Kidney Disease, Conservative Management, Signs and Symptoms, Renal Dialysis, Palliative Care, Insuficiência Renal Crônica, Tratamento Conservador, Sinais e Sintomas, Diálise Renal, Cuidados Paliativos

## Abstract

Estimates suggest that 20-30% of the deaths of patients with chronic kidney disease with indication to undergo dialysis occur after refusal to continue dialysis, discontinuation of dialysis or inability to offer dialysis on account of local conditions. Contributing factors include aging, increased comorbidity associated with chronic kidney disease, and socioeconomic status. In several occasions nephrologists will intervene, but at times general practitioners or family physicians are on their own. Knowledge of the main etiologies of chronic kidney disease and the metabolic alterations and symptoms associated to end-stage renal disease is an important element in providing patients with good palliative care. This review aimed to familiarize members of multidisciplinary care teams with the metabolic alterations and symptoms arising from chronic kidney disease treated clinically without the aid of dialysis.

## RESUMO

Estimativas sugerem que em torno de 20% a 30% dos óbitos de pacientes com doença renal crônica em estágio dialítico decorrem de renúncia à diálise, da interrupção do tratamento dialítico ou da incapacidade de oferecer tratamento dialítico em função das condições locais. O envelhecimento populacional, o aumento das comorbidades associadas à doença renal crônica e o nível socioeconômico do país contribuem para esse cenário. Em diversas ocasiões o nefrologista estará presente para intervir ativamente, mas em outras o médico generalista ou o médico de família estarão sós. O conhecimento das principais etiologias da doença renal crônica e das alterações metabólicas e dos sintomas associados à fase terminal da doença renal são condições importantes para a evolução do paciente sem sofrimento para uma boa morte. O objetivo desta revisão é familiarizar os membros da equipe multiprofissional para o reconhecimento e conduta quanto às alterações metabólicas e aos sintomas decorrentes da doença renal crônica tratada clinicamente sem suporte dialítico.

## INTRODUCTION

The number of patients with chronic kidney disease (CKD) on dialysis has increased substantially within the last two decades as a consequence of aging and the elevated prevalence of diabetes *mellitus* and systemic hypertension.^1^ Consequently, the proportion of patients with multiple comorbidities on dialysis has also increased.

Today, individuals with coronary artery disease, chronic obstructive pulmonary disease, solid tumors, severe peripheral artery disease, severe cognitive impairment or marked mobility limitations are often accepted in ambulatory dialysis programs.^2^ Dialysis may deteriorate the clinical condition of patients in this situation, along with their own quality of life and that of their families.^3^^-^ 5

On the other hand, the proportion of health funds assigned to the treatment of these patients has grown steadily and compromised the economic stability of the healthcare system. This issue affects developing and developed nations.

The imminent collapse of healthcare has led health authorities in Brazil^6^ and other countries to develop programs to enable the early diagnosis of chronic kidney disease and the referral of patients in need of dialysis to medical care in order to delay the progression to end-stage renal disease.

Once the diagnosis of CKD has been established, it is up to the physicians to delay the progression of the disease and to discuss with patients and their families the best course of therapy for the final stages of renal disease: dialysis, transplantation or conservative management without dialysis.

The trend of offering dialysis to older patients with multiple comorbidities has stirred interest around alternative therapies that do not involve dialysis or organ transplantation, particularly for individuals older than 70 years.^7^^-^ 9

Several guidelines have established that patients with CKD stage 4 and glomerular filtration rates (GFR) ranging between 29 and 15 ml/min/1.73m^2^ and with CKD stage 5 and GFR below 15 ml/min/1.73m^2^ should be supervised by a nephrologist.^10^^,^ 11 The earlier a nephrologist is involved, the greater the chances of delaying the progression of disease and the start of dialysis.^12^^,^ 13

On the other hand, when the option is made for conservative management or discontinuation of dialysis, the opportunity of involving specialized palliative care and end-of-life support professionals arises.^14^ Therefore, in this stage of the disease, general practitioners, family physicians, nephrologists, palliative care physicians and a multidisciplinary team featuring nutritionists, physical therapists, social workers, and spiritual advisors may work in concert to aid the patients.^15^

## THE ROLE OF NEPHROLOGISTS

Every patient with a GFR below 30 ml/min/1.73m^2^ should be followed by a nephrologist, since at this level of renal function the metabolic alterations secondary to CKD become noticeable.^10^^,^ 11 However, the frequency and intensity of symptoms vary significantly from one individual to the next. Patients with end-stage renal disease often arrive at emergency units not knowing they have kidney disease.^16^ These cases show the ability our bodies have to adapt to the metabolic changes introduced by CKD without producing obvious symptoms. When the choice is made for not undergoing dialysis, nephrologists must take advantage of this trait to manage their patients conservatively until the advanced stages of the disease and the death of the patient.

## ETIOLOGIC DIAGNOSIS OF CHRONIC KIDNEY DISEASE

From the standpoint of public health policy, systemic hypertension and diabetes *mellitus* rank as the main etiologies of CKD.^1^ Other conditions such as glomerulonephritis, urinary tract malformations, and inherited renal diseases are much less frequent.

Clinical trials have shown that managing hypertension and diabetes produces a significant impact on the progression of renal disease. Most of the guidelines have indicated that blood pressure should be maintained below 130/80 mmHg and glycated hemoglobin below 7% to delay the progression of CKD.^10^^,^ 11 ^,^ 17 Therefore, effort must be made to control these parameters, particularly when the GFR drops to less than 30 ml/min/1.73m^2^. However, excessive decreases in blood pressure and glucose levels may be linked with severe complication. It should be noted that in the two conditions there is no association between intensity of symptoms and progression of kidney disease.

Inhibitors of the renin-angiotensin system and angiotensin II receptor blockers have been used to decrease hyperflow, intraglomerular hypertension, proteinuria, and systemic blood pressure, consequently delaying GFR decreases.^18^^-^ 22 Drugs in these classes are prescribed in the early stages of CKD, particularly to individuals with diabetes. However, in the more advanced stages of CKD when the GFR is below 12-15 ml/min/1.73m^2^, they may be suspended to allow improvements in renal function through the reestablishment of hyperflow and intraglomerular hypertension.^22^ This strategy is particularly useful when the goal is to delay the start of dialysis or when the patient chooses to waive dialysis in favor of conservative management.^23^

Approaches to manage diabetes vary. Patients given oral hypoglycemic drugs are usually given insulin when their GFR drops to levels below 30 ml/min/1.73m^2^, and new drugs with enhanced safety characteristics for patients with CKD have been introduced.^10^^,^ 11 ^,^ 17 Individuals previously on insulin therapy are often required to decrease dosages, since insulin is metabolized in the renal tubular cells and has its half-life prolonged in the advanced stages of kidney disease.^10^^,^ 11 ^,^ 17

## MANAGING METABOLIC ALTERATIONS IN PATIENTS REFUSING DIALYSIS

The progression of CKD is tied to the deterioration of metabolic parameters and consequently to the onset of symptoms.^24^^-^ 26 Interventions aimed at correcting metabolic alterations may reduce symptoms and improve the quality of life of patients off dialysis.

### DIET

The kidneys are natural filters; therefore, the lower the load generated by metabolic processes the less the kidneys have to work, the easier the maintenance of metabolic balance, and the lower the intensity of symptoms. Regardless of the belief that protein restriction slows the progression of CKD, adjusting patient diet to the stages of kidney disease is a powerful tool to manage the symptoms of CKD.^27^

Excessive protein intake - animal protein in particular - overloads the kidneys, decreases the renal functional reserve, and accelerates the progression of the disease.^10^^,^ 11 Protein metabolism produces large amounts of fixed acids that have to be buffered, thus rising the consumption of bicarbonate and exacerbating metabolic acidosis, the latter closely tied to gastrointestinal symptoms such as nausea, vomiting, and anorexia.

Protein restriction at levels around 0.8 to 1 g/kg/day is well tolerated and effective in managing metabolic alterations when the GFR resides between 10 and 30 ml/min/1.73m^2^. When the GFR lies between 5 and 10 ml/min/1.73m^2^, the management of symptoms requires more intense protein restriction, in the order of 0.6 to 0.8 g/kg/day. Oligosymptomatic patients with a GFR under 5 ml/min/1.73m^2^ must reduce protein intake to less than 0.5 g/kg/day.^10^^,^ 11 ^,^ 28 In this scenario, in order to prevent the development or worsening of malnutrition, when protein intake is below 0.3 g/kg/day patients are required to supplement their diets with keto-analogues to preserve their nutritional status, decrease protein catabolism, and reestablish the acid-base balance.^27^^-^ 29 In short, protein restriction with or without keto-analogue supplementation may be considered for patients with CKD refusing or delaying the start of dialysis, since the risk of protein/energy malnutrition is minimal when patients are carefully selected, properly followed, and rigorously advised when it comes to their nutrition.^29^^,^ 30

In addition to protein restriction, patients are often required to decrease the intake of salt to manage hypertension and edema and to limit the ingestion of certain fruits, vegetables, and greens to control serum potassium levels.^31^ Patients required to endure multiple limitations tend to comply less with treatment, and physicians have to analyze each case individually to stress and prioritize the more relevant aspects of diet restrictions.^31^ Nutritionists play a key role in the successful implementation of this strategy.

Protein restriction has not been frequently used in clinical practice. Western eating habits are known for elevated intakes of protein and salt, and attempts to decrease the consumption of the two are not welcome by patients, who often feel stigmatized. Nevertheless, uremia tends to induce patients to spontaneously decrease the intake of protein, leaving little room for medical advice in this direction.^32^ However, when the option is made for conservative management without dialysis, protein and salt restriction combined with supplementation with keto-analogues is fundamentally needed to control one's nutritional status, fluid and electrolyte balance, and uremia symptoms.

### ANEMIA

The progression of kidney disease is linked to the gradual deterioration of hemoglobin levels, particularly when the GFR is below 30 ml/min/1.73m^2^. The management of anemia through iron replacement accompanied or not by erythropoietin is a powerful tool in providing relief against symptoms such as lethargy, fatigue, and inability to focus.^33^ The guidelines have recommended that hemoglobin levels be kept between 10 and 12 g/dl^34^; however, levels in the range of 12 to 13 g/dl are more effective in managing symptoms and have been associated with improved quality of life, two important variables in conservative care without dialysis.^35^

### MINERAL AND BONE METABOLISM

As CKD progresses, alterations in mineral and bone metabolism become apparent when the GFR reaches values below 50 ml/min/1.73m^2^.^36^ At first there is a decrease in the synthesis of vitamin D, which leads to decreased calcium intestinal absorption, proneness to hypocalcemia, and stimulation of parathyroid hormone secretion. These alterations become more severe with concomitant increases in serum phosphorus levels when the GFR is below 15 ml/min/1.73m^2^.

The management of mineral and bone metabolism in individuals with CKD includes vitamin D replacement, oral supplementation of calcium, and, in more advanced disease, the administration of intestinal phosphate binders.^36^ These measures aim to increase the intestinal absorption of calcium, address hypocalcemia, prevent hyperphosphatemia, and manage the progressive increase of parathyroid hormone levels. However, these drugs accentuate the effects of polypharmacy seen in patients with CKD, while phosphate binders introduce predominantly gastrointestinal side effects.

The prescription of drugs to delay the progression of osteodystrophy in patients with a GFR below 10 ml/min/1.73m^2^ refusing dialysis seems pointless, since progression to death occurs at a faster pace. However, in cases of symptomatic hypocalcemia, replacement of calcium and vitamin D is mandatory.^36^

### EDEMA

In the progression of CKD, managing fluid and electrolyte balance is relevant not only to maintain safe blood pressure levels, but also to control edema. Edema is quite frequent in the more advanced stages of CKD, mainly in patients having trouble complying with dietary and salt restrictions. The prescription of loop diuretics (oral furosemide 80 to 240 mg/day) combined or not with thiazide diuretics (oral hydrochlorothiazide or chlorthalidone 25 to 50 mg/day) is a powerful tool to manage edema and decrease blood pressure levels.^37^^,^ 38 However, imprudent use of diuretics may cause hypovolemia, intensification of cramps, hypo- or hypernatremia, hypokalemia, and impaired management of calcemia.^39^ Potassium-sparing diuretics and renin inhibitors must be prescribed cautiously, since they favor the development of hyperkalemia.^39^

### METABOLIC ACIDOSIS

Metabolic acidosis is a very frequent complication when the GFR is below 30 ml/min/1.73m^2^, particularly in patients with tubulointerstitial disease. Patients failing to comply with dietary protein restrictions tend to suffer with more intense manifestations of metabolic acidosis.^40^

Acidosis in CKD worsens inappetence, strengthens gastrointestinal symptoms such as nausea and vomiting, and intensifies feelings of dyspnea.^41^ Treating acidosis with oral bicarbonate (500 to 1000 mg, 3 to 4 times a day) improves several symptoms attributed to uremia, but higher doses may contribute to the appearance or worsening of edema and the onset of abdominal colic and diarrhea. The goal is to keep serum bicarbonate levels above 22 mEq/L.^42^ Patients with severe acidosis and untreatable nausea might require intravenous bicarbonate injections.

### HYPERKALEMIA

Hyperpotassemia is a frequent complication in patients with a GFR below 15 ml/min/1.73m^2^ managed conservatively. The prescription of drugs such as angiotensin-converting-enzyme (ACE) inhibitors, angiotensin II receptor blockers (ARBs), potassium-sparing diuretics, and renin inhibitors tends to intensify hyperkalemia. In addition, patients failing to comply with dietary recommendations and individuals with severe metabolic acidosis are particularly prone to developing hyperpotassemia.^43^

In such conditions, potassium levels are managed by restricting the prescription of drugs that decrease the renal excretion of potassium, controlling metabolic acidosis and, in selected cases, prescribing ion-exchange resins that adsorb potassium in the digestive tube in exchange for calcium or sodium.^44^

### SYMPTOMS AND QUALITY OF LIFE OF PATIENTS REFUSING DIALYSIS

In addition to maintaining the metabolic equilibrium, the kidneys play an important role as hormone-producing organs. The symptoms presented by the patients and the changes in quality of life derive from the progressive loss of these two functions.

Patients with CKD stage 5 off dialysis and on conservative management present a mean number of nine symptoms.^5^^,^ 8 Although they vary in intensity, symptoms become more intense with the progression of renal failure and the proximity of death.^45^ Despite the significant occurrence of symptoms in patients managed conservatively without dialysis, few studies have compared the quality of life of these individuals versus patients on dialysis by medical recommendation. Existing reports tell of similar outcomes.^5^^,^ 46

The most common complaints made by patients managed conservatively include weakness, malaise, lethargy, prostration, somnolence, trouble sleeping, attention deficit, depression, inappetence, dry mouth, metallic taste in the mouth, nausea, pruritus, dry skin, dyspnea, edema, cramps, restless legs syndrome, and pain.^24^^-^ 26

### CLINICAL MANAGEMENT OF SYMPTOMS OF PATIENTS REFUSING DIALYSIS

It is important to know the profile of symptoms of patients managed conservatively, since in this stage of the disease the focus shifts from delaying the progression of renal failure and the start of dialysis to managing symptoms.

### PAIN

Pain of any kind or intensity is reported by 60% of the patients with CKD treated conservatively.^8^ Recognizing, managing, and alleviating pain is of the utmost importance, in addition to helping patients cope with symptoms such as anxiety, psychomotor agitation, dyspnea, restless legs syndrome, and depression.^47^

Anti-inflammatory drugs are contraindicated to manage pain in patients with CKD since they have been associated with deterioration of renal function and gastrointestinal bleeding.^48^ Paracetamol and dipyrone may be prescribed safely to patients with CKD. Codeine and its derivatives should be avoided, since their metabolization produces active substances such as morphine and morphine derivatives, known to favor respiratory depression and somnolence. Tramadol is safer than codeine, although it has also been associated with respiratory depression. Morphine and diamorphine may cause respiratory depression and somnolence and should be avoided in patients with a GFR below 30 ml/min/1.73m^2^. Oxycodone seems to be less toxic than morphine, but should be prescribed only in short-course preparations at lower dosages and longer administration intervals. Methadone is relatively safe, since it is metabolized in the liver and the accumulation of metabolites is minimal in patients with CKD.^24^^-^ 26

**Figure 1:**
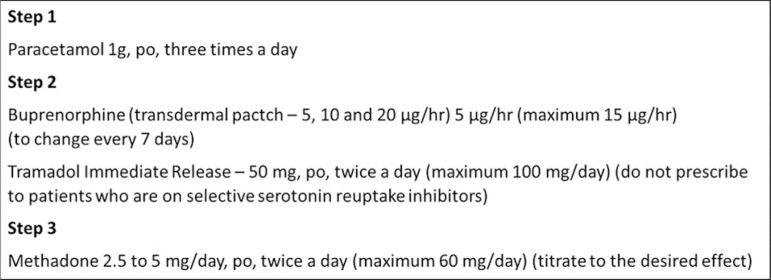
Analgesia for patients with advanced chronic kidney disease.

The Figure 1 shows an analgesia scheme to patients with advanced CKD.

Patients with neuropathic pain may take gabapentin (100 mg, oral, at night) or pregabalin (25 mg, oral, twice a day), although somnolence and dizziness may require dosage reductions. An alternative is amitriptyline (10 mg, oral, at night, maximum dose of 100 mg), but it should be avoided in patients with heart disease and at risk for arrhythmia.^25^

### NAUSEA AND VOMITING

More than half of the patients with CKD who refuse dialysis suffer with nausea and vomiting.^8^ Establishing symptom etiology is an important step in defining the treatment. Metabolic acidosis and diabetic gastroparesis are frequent causes of nausea and vomiting, but terminal uremia *per se* might cause persistent nausea, which gets worse as death is closer. Therefore, every patient with CKD in the process of dying should be offered antiemetic drugs.

Haloperidol is the drug of choice to manage nausea associated with uremia. In addition to being an excellent antiemetic, it also has anxiolytic properties. When needed, the usual initial dose is 1.5 mg - administered subcutaneously or orally - and the maximum dose is 10 mg/day.^24^ Subcutaneous metoclopramide 10 mg, three times a day, at a maximum of 60 mg/day, may be attempted in cases of stasis or obstruction impeding gastric emptying, a frequent event in diabetic patients.^24^^,^ 25 Dose reductions may be required, since metoclopramide may accumulate and produce extrapyramidal side effects.

### PRURITUS

Pruritus is reported by 65% of nondialytic patients with CKD stage 5.^8^ Its multifactorial pathogenesis involves factors such as disorders of calcium, phosphate, and parathyroid hormone metabolism, accumulation of uremic toxins, systemic inflammation, and dry skin.^49^

There is no evidence to support any particular course of treatment.^25^^,^ 26 Water-based softening creams twice or three times a day might provide some relief. Antihistamines do not decrease uremic pruritus, but their sedative properties might help with sleep disorders. Gabapentin may be administered under close surveillance, since accumulation of the drug in individuals with renal disease might induce somnolence. Mirtazapine, a noradrenalin receptor inhibitor, alleviates pruritus possibly by decreasing central sensitization to itches.

### DYSPNEA

Dyspnea is present in the complaints of 65% of the patients with indication for dialysis managed conservatively.^8^ Dyspnea associated with uremia is a multifactorial event that may involve anemia, hypertensive and ischemic cardiomyopathy, fluid overload, pulmonary edema, and metabolic acidosis.^50^

Anemia may be managed by ensuring the proper metabolism of iron and with the administration of erythropoietin; blood transfusions may be performed in selected cases. Careful use of high-dose diuretic therapy helps control fluid overload, particularly when performed concomitantly with restrictions to water and salt intake. The correction of metabolic acidosis with bicarbonate replacement decreases the importance of the respiratory buffer system and consequently of the feeling of dyspnea. Patients with significant anxiety may benefit from the prescription of low-dose therapy with benzodiazepines, midazolam or opioids.^24^^,^ 25 Patients on these drugs have to be closely monitored to avoid toxicity and excessive somnolence. Patients with hypoxia may be offered intermittent or continuous administration of oxygen. Non-pharmacological approaches such as ventilators, room air circulation, respiratory physiotherapy, and occupational therapy may provide relief against dyspnea.

### RESPIRATORY SECRETIONS

The accumulation of secretions in the respiratory tract is a frequent complication among individuals with CKD reaching the end of their lives. Hydration control decreases the production of secretions and reduces patient discomfort. Positioning the patient properly helps mobilize secretions and facilitates oral hygiene. Oral N-Butylscopolamine bromide 20 mg on demand or 60 mg in continuous subcutaneous infusion using an infusion pump in quantities titrated up to 180 mg/day decreases the production of secretions in the respiratory tract.^24^

### RESTLESS LEGS SYNDROME

When properly investigated, restless legs syndrome (RLS) is found in up to 50% of the patients with advanced CKD treated conservatively.^8^ The syndrome is characterized by the urge and involuntary need to move the legs, often accompanied by a feeling of discomfort in the lower limbs. Manifestations tend to worsen with rest and at night and to improve with physical activity.

The pathophysiology of RLS has not been completely elucidated and apparently involves dopaminergic pathways in the central nervous system. Gabapentin (initial dose 300 mg/day) and dopamine agonists such as pramipexole (initial dose 0.125 mg/day) are effective at managing the syndrome. Clonazepam (initial dose 0.5 to 1 mg/day) may be useful, but has been associated with increased somnolence.^51^

### ANXIETY AND AGITATION

Although anxiety and agitation are often seen in patients with advanced CKD, the exact prevalence of these findings is unknown. Potential and reversible causes of anxiety and agitation include pain, urinary retention, obstipation, irritability, and side effects from medication, mainly corticosteroids. Anxiolytics such as lorazepam or diazepam may be prescribed to manage symptoms. Subcutaneous midazolam 10-30 mg in 24 hours administered with an infusion pump is an alternative for more severe cases; additional doses of 2.5 to 5 mg may be administered as salvage therapy.^24^^,^ 25

### DEPRESSION

Although diagnosis varies between diagnostic instruments, it has been estimated that 20% of patients with CKD suffer from depression.^52^ There is a significant overlap between the symptoms of depression and CKD, a factor that contributes to the first being underdiagnosed and undertreated.^53^ Antidepressants are the basis for treatment. Sertraline 50 to 200 mg once a day or fluoxetine 20 to 60 mg once a day are safe medications and do not require dose adjustments.^53^ Although cognitive-behavioral therapy has gained attention in the treatment of depression among patients with CKD, there is no study on its validity for patients refusing dialysis.^54^ Nonetheless, support by a multidisciplinary team and proper management of metabolic alterations and symptoms caused by refusal to undergo dialysis help decrease the number of cases of severe depression.

## CONCLUSIONS

While some authors have reported little or minimal improvement in the survival of elderly patients with multiple comorbidities and ischemic heart disease offered dialysis,^15^^,^ 55 ^,^ 56 others have described improvements in survival with dialysis.^3^^,^ 57 ^,^ 58 However, these improvements in survival are followed by complications such as infection, deterioration of comorbidities, hemodynamic instability, troubles with the vascular access, travels to the dialysis center, and frequent prolonged hospitalization. And these complications lead to the deterioration of quality of life, since patients end up spending a significant portion of their time dealing with medical problems.

With this context in mind, many patients have refused dialysis with the support of their families and physicians.^59^^,^ 60 Patients refusing dialysis have their functions and symptoms relatively under control until two or three months before they die. It is the task of the physician and the multidisciplinary care team to anticipate and identify symptoms and provide the relief needed.
